# Squalenoylated Nanoparticle Pro-Drugs of Adjuvant Antitumor 11α-Hydroxyecdysteroid 2,3-Acetonides Act as Cytoprotective Agents Against Doxorubicin and Paclitaxel

**DOI:** 10.3389/fphar.2020.552088

**Published:** 2020-09-11

**Authors:** Máté Vágvölgyi, Péter Bélteky, Dóra Bogdán, Márta Nové, Gabriella Spengler, Ahmed D. Latif, István Zupkó, Tamás Gáti, Gábor Tóth, Zoltán Kónya, Attila Hunyadi

**Affiliations:** ^1^ Institute of Pharmacognosy, Interdisciplinary Excellence Centre, University of Szeged, Szeged, Hungary; ^2^ Department of Applied and Environmental Chemistry, Interdisciplinary Excellence Centre, University of Szeged, Szeged, Hungary; ^3^ Department of Organic Chemistry, Semmelweis University, Budapest, Hungary; ^4^ Institute of Materials and Environmental Chemistry, Research Centre for Natural Sciences, Budapest, Hungary; ^5^ Department of Medical Microbiology and Immunobiology, University of Szeged, Szeged, Hungary; ^6^ Department of Pharmacodynamics and Biopharmacy, Faculty of Pharmacy, University of Szeged, Szeged, Hungary; ^7^ Interdisciplinary Centre for Natural Products, University of Szeged, Szeged, Hungary; ^8^ Servier Research Institute of Medicinal Chemistry (SRIMC), Budapest, Hungary; ^9^ NMR Group, Department of Inorganic and Analytical Chemistry, Budapest University of Technology and Economics, Budapest, Hungary; ^10^ MTA-SZTE Reaction Kinetics and Surface Chemistry Research Group, University of Szeged, Szeged, Hungary

**Keywords:** ecdysteroid, squalene nanoparticle pro-drug, self-assembly, low-density lipoprotein targeting, cancer, multi-drug resistance

## Abstract

Several ecdysteroid acetonides act as adjuvant chemo-sensitizing agents against various cancer cell lines, and they can be formulated to self-assembling nanoparticle (NP) pro-drugs through a hydrolysable conjugation with squalene. In the bloodstream such squalenoylated nanoparticles dissolve into low-density lipoprotein (LDL) that allows targeting tissues containing high levels of LDL-receptors. In this work, ajugasterone C 2,3;20,22-diacetonide (**3**) and 11α-hydroxypoststerone 2,3-acetonide (**4**) were squalenoylated to obtain two new ecdysteroid pro-drugs (**6** and **7**) and their nano-assemblies (**6_NP_** and **7_NP_**). A complete NMR signal assignment of **6** and **7** was achieved. Interaction of compounds **3** and **4** with chemotherapeutics was studied by the Chou-Talalay method. Compound **3** showed strong synergism with doxorubicin on a multi-drug resistant lymphoma cell line. In contrast, its nanoassembly **6_NP_** significantly decreased the cytotoxicity of doxorubicin on these MDR cells, strongly suggesting that at least the 2,3-acetonide group was cleaved by the acidic pH of lysosomes after endocytosis of the prodrug. Further, compound **4** acted in strong antagonism with paclitaxel on MCF-7 cells and its nanoassemby **7_NP_** also protected MCF-7 cells from the effect of paclitaxel. Our results suggest that acid-resistant A-ring substitution would be crucial to design adjuvant antitumor squalenoylated ecdysteroid prodrugs. Additionally, our results may be considered as a serendipitous discovery of a novel way to deliver cytoprotective, adaptogen ecdysteroids to healthy tissues with upregulated LDL-R.

## Introduction

Nanotechnology offers various perspectives to overcome the insufficient therapeutic efficacy of a biomolecule. Over the previous decades, a wide array of nano-sized delivery platforms had been developed in antitumor chemotherapy for a more efficient delivery of chemotherapeutic agents to the target cells (Li et al., 2017). Typically, the use of nanomaterials as *in vivo* transport systems serves more than one intention: a) by placing the drug in a biocompatible, protective matrix, we can modulate its physicochemical stability in the organism, therefore improving its pharmacokinetic and pharmacological properties, and b) by considering the tissue- or cell-specific delivery mechanism of nanoparticles (NPs) we can have a partial control over the accumulation and distribution of the drug, and, accordingly, directly or indirectly target the tumor (Xu et al., 2015; Shi et al., 2017).

One particularly simple, yet efficient way to design anticancer NPs is the preparation of self-assembling drug conjugates (Fumagalli et al., 2016). We can obtain these molecules through synthesis, by chemically linking the chosen chemotherapeutic agent to an essential functional unit, the so-called inducer. Organic molecules, such as terpenes, polysaccharides, or polymeric chains with high biocompatibility, are widely used as inducers, which can spontaneously self-assemble into NPs in an aqueous medium without the need for using any additional surfactants to stabilize their colloid suspensions (Bildstein et al., 2011; Delplace et al., 2014). In some instances, connecting the drug and the inducer can be achieved by using a so-called linker moiety, which may further enhance the *in vivo* release of the therapeutic agent from its conjugate (Borrelli et al., 2014). During the synthetic preparation of bioconjugates, components are coupled together through covalent bonds that are hydrolysable in a biological environment (e.g., esters), so that such self-assembled NPs will typically act as pro-drugs (Cheetham et al., 2017).

Among the possible inducers, squalene is a particularly attractive choice due to its biocompatibility, inertness, and the ease of transforming the terminal double bond to versatile functional groups that may be linked to the drug (Desmaele et al., 2012; Maksimenko et al., 2014). It has recently been revealed that NPs of squalene conjugates do not remain intact after getting into the bloodstream. Instead, they get dissolved into lipoproteins (whose most affected fraction depends on the species, in humans it is mainly low-density lipoprotein or rather LDL) that will transport them in the bloodstream, and this allows a unique targeting of cancer cells that commonly display high expression and activity of LDL receptors (LDL-R) (Sobot et al., 2017).

Ecdysteroids, analogs of the insect molting hormone ecdysone, exert a wide range of bioactivities in mammals (Lafont and Dinan, 2003; Dinan et al., 2020). We have previously reported that less polar ecdysteroid derivatives can strongly sensitize both multi-drug resistant (MDR) and drug-susceptible cancer cell lines to various chemotherapeutics (Martins et al., 2012; Martins et al., 2015). We have been exploring relevant structure-activity relationships over the years, and found that the most important is the presence of a 2,3-acetonide group (Hunyadi et al., 2017). This moiety is unfortunately also the most chemically sensitive part of these compounds, particularly in acidic environment. Therefore, nano-formulation of these compounds can not only serve as a relevant targeting strategy, but it may also significantly increase their stability. Previously, we reported the preparation and evaluation of squalene-conjugates obtained from poststerone 2,3-acetonide 20-oxime (Bogdan et al., 2018). These compounds, together with 25-squalenoylated analogs of 20-hydroxyecdysone 2,3;20,22-diacetonide (20DA), were subsequently formulated into doxorubicin-containing hetero-nanoparticles that could efficiently inhibit proliferation of adriamycin-resistant A2780_ADR_ cancer cells (Fumagalli et al., 2018).

In the present work, we aimed to further expand the available chemical and pharmacological space by preparing further self-assembling conjugates and NPs from other, less abundant ecdysteroids containing an 11α-OH group for a convenient ester coupling, and to evaluate their effect on the chemoresistance of MDR and non-MDR cancer cell lines.

## Materials and Methods

### Materials

Reagents and solvents were purchased from Sigma (Merck KGaA, Darmstadt, Germany) and were applied for the corresponding research purpose without any further purification. Each synthetic reaction was monitored by thin-layer chromatography (TLC) on Kieselgel 60F_254_ silica plates (Merck KGaA, Darmstadt, Germany). The characteristic spots of materials were examined under UV illumination at 254 and 366 nm. Ajugasterone C (**1**) was previously isolated from *Serratula* *wolffii* (Hunyadi et al., 2007).

### Synthetic Procedures

#### Synthesis of Compound 2

Compound **2** was prepared according to our previously published method (Issaadi et al., 2019). Briefly, a 1 g aliquot of ajugasterone C (**1**) was dissolved in 80 ml of methanol. One equivalent of (diacetoxyiodo)benzene (PIDA) was added to the solution, and subsequently, the reaction mixture was left under stirring for 45 min at RT. Following this, the solution was neutralized with 10% aq. NaHCO_3_ and then, the solvent was evaporated on a rotary evaporator. The obtained dry residue was re-dissolved in methanol, and silica gel (~4 g) was added to the solution. After this, the solvent was removed under reduced pressure allowing the preparation of the sample for dry loading flash chromatographic purification. This resulted in the successful isolation of compound **2** (0.59 g, 74.8%).

#### Synthesis of Compounds 3 and 4

Derivatization of the diols of ecdysteroids **1** and **2** was performed as published before (Martins et al., 2012). Briefly, a 500 mg aliquot of the corresponding substrate was dissolved in a concentration of 1 g/100 ml (50 ml) in acetone. To the obtained solution, 500 mg of phosphomolybdic acid (PMA) hydrate was added, and the mixture was sonicated for 25 min at RT. Subsequently, the solution was neutralized with 10% aq. NaHCO_3_ and then, the solvent was evaporated under reduced pressure. The obtained products were extracted from their aqueous residue using ethyl acetate (3 ml x 100 ml), and the organic fractions were combined and dried over Na_2_SO_4_. Following this, the drying agent was removed by filtration, and the solvent was evaporated on a rotary evaporator. The residue was subjected to flash chromatographic purification to yield ajugasterone C 2,3;20,22-diacetonide (**3**; 364 mg, 62.4%), or 11α-hydroxypoststerone 2,3-acetonide (**4**; 386 mg, 69.8%) in pure form.

#### Synthesis of Ecdysteroid Conjugates 6 and 7

Self-assembly inducer squalene was functionalized and conjugated with sebacic acid following previously published procedures, which had allowed us the preparation of compound **5** (Borrelli et al., 2014; Maksimenko et al., 2014). An aliquot of 0.2 mmol of ecdysteroid 3 (112.2 mg) or 4 (83.7 mg) and 142.7 mg of compound **5** (0.25 mmol, 1.25 equiv.) were dissolved in 10 ml of dry dichloromethane in a two-neck round-bottom flask. Later, 48.9 mg of 4-dimethylaminopyridine (DMAP; 0.4 mmol, two equiv.) and 115 mg of (3-dimethylaminopropyl)-N′-ethylcarbodiimide hydrochloride (EDC·HCl; 0.6 mmol, three equiv.) were added to the solution, and then, the reaction mixture was stirred under an argon atmosphere at RT for overnight. Following this, the solution was neutralized with 10% aq. NaHCO_3_ and was diluted with brine (30 ml) allowing to perform an extraction with dichloromethane (4 ml x 30 ml). The collected organic fractions were combined and dried under Na_2_SO_4_, and subsequently, the drying agent was removed through filtration. The solvent of the sample was evaporated, and then, the residue was purified utilizing preparative NP-HPLC affording the isolation of compound **6** (180.9 mg, 81.2%) or the side chain-cleaved conjugate analogue **7** (97.9 mg, 58.9%), as a colorless oil.

### Chromatographic Conditions

Following organic synthesis, chromatographic purification of substances was carried out on either a Combiflash Rf+ flash chromatographic instrument (TELEDYNE Isco, Lincoln, NE, USA) equipped with DAD-ELS detection using commercially available RediSep columns (TELEDYNE Isco, USA), or on an Armen Spot Prep II preparative HPLC purification system (Gilson, Middleton, WI, USA) equipped with a dual-wavelength UV-VIS detector. Flash chromatographic purifications were performed on 4–24 G silica columns with adequately chosen eluent ratios of dichloromethane—methanol. For preparative HPLC separations, a Phenomenex Luna^®^ 5 µm Silica (2) 100 Å 250 mm x 21.2 mm column (Phenomenex Inc., Torrance, CA, USA) was used, typically in isocratic elution mode with adequately chosen eluent ratios of cyclohexane—isopropanol. In general, the applied flow rates were 15 ml/min, and the wavelengths of detection were 210 andd 250 nm. Purity of the isolated compounds was determined on a Jasco HPLC instrument (Jasco International Co. Ltd., Hachioji, Tokyo, Japan) equipped with LC capillary cables proper for analytical purposes. The analysis was carried out on a 250 mm x 4.6 mm analogue of the preparative silica column using the peak area % data of the PDA chromatogram recorded between 210 and 410 nm. During the analytical-scale HPLC measurements, the applied flow rate was a constant 1 ml/min.

### Structure Elucidation

The compounds’ mass spectra were recorded on an Agilent 1100 LC-MS instrument (Agilent Technologies, Santa Clara, CA, USA) coupled with Thermo Q-Exactive Plus orbitrap analyzer (Thermo Fisher Scientific, Waltham, MA, USA) used in positive mode.

^1^H (800 and 500 MHz) and ^13^C (200 and 125 MHz) NMR spectra were recorded at room temperature on Bruker Avance III spectrometers equipped with cryo probeheads. Amounts of approximately 3–5 mg of compounds were dissolved in 0.6 ml of chloroform-*d* and transferred to 5 mm NMR sample tubes. Chemical shifts are given on the *δ*-scale and are referenced to the solvent chloroform-*d*: *δ*_C_ = 77.00 and *δ*_H_ = 7.27 ppm). Pulse programs of all experiments (^1^H, ^13^C, DEPTQ, 1D sel-TOCSY, gs-HSQC, and gs-HMBC (optimized for 8 and 10 Hz respectively), band–selective–HSQC, –HMBC and HSQC–TOCSY were taken from the Bruker software library. For 1D measurements, 64K data points were used to yield the FID. For 2D measurements, on the 500 MHz spectrometer in case of the HSQC spectrum data points (t_2_ x t_1_) were acquired with 2 K x 128, in case of the HMBC spectrum data points (t_2_ x t_1_) were acquired with 2 K x 256, respectively. In the band-selective HMBC experiment, the used digital resolution was 1.04 Hz per point; in the band-selective HSQC experiment it was 0.13 Hz per point. For F_1_ linear prediction was applied to enhance the resolution. Most ^1^H assignments were accomplished using general knowledge of chemical shift dispersion with the aid of the proton-proton coupling pattern (^1^H NMR spectra). The NMR signals of the products were assigned by comprehensive one- and two-dimensional NMR methods using widely accepted strategies (Duddeck et al., 1998; Pretsch et al., 2002).The ^1^H and ^13^C NMR data for the steroid moiety of compounds **6** and **7**, are compiled in **Table 1**, whereas the signals of the R-groups are summarized in **Table 2**. The characteristic NMR and HRMS spectra of compounds **6** and **7** are presented as supporting information.

**Table 1:** ^1^H and ^13^C chemical shifts, multiplicities and characteristic coupling constants (Hz) of the steroid moiety of compounds **6** and **7** in CDCl_3_.

Atom no.	6^b^			7^a^		
	H	*J* (Hz)	C	H	*J* (Hz)	C
1β	1.20 dd	13.9; 10.7	39.85	1.21 dd	13.9; 10.9	39.88
1α	1.83 dd	13.9; 6.1		1.83 dd	13.9; 6.1	
2	4.42 ddd	10.7; 6.1; 4.5	72.31	4.44 ddd	10.9; 6.1; 4.5	72.26
3	4.31 dd	4.5; 4.5	71.51	4.31 ddd	4.5; 4.5; 1.5	71.48
4β	2.16 m		27.11	2.15 m		27.07
4α	1.80 m			1.78 ddd	17.5; 13.4; 4.5	
5	2.33 m		52.06	2.35 m		52.07
6	–		202.26	–		201.90
7	5.89 d	2.9	122.73	5.89 dd	2.9; 1.0	123.22
8	–		159.52	–		158.22
9	3.13 dd	9.1; 2.9	38.52	3.18 dd	9.1; 2.9	38.59
10	–		38.57	–		38.63
11β	5.31 ddd	10.4; 9.1; 6.5	70.61	5.30 ddd	10.4; 9.1; 6.5	70.21
11α	–			–		
12β	2.01 m		37.18	2.29 m		36.11
12α	2.31 m			2.25 m		
13	–		46.88	–		46.95
14	–		84.42	–		84.20
15β	2.08 m		31.84	2.10 m		32.15
15α	1.55 m			1.65 m		
16β	2.08 m		21.11	2.33 m		21.10
16α	1.87 m			1.93 m		
17	2.24 dd	10.0; 7.9	48.84	3.29 dd	9.7; 7.9	58.18
18	0.85 s		17.66	0.68 s		17.60
19	1.03 s		23.53	1.03 s		23.55
20	–		83.77	–		208.39
21	1.14 s		21.83	2.15 s		31.44
22	3.59 dd	9.2; 2.5	81.58			
23	1.45/1.35 m		26.78			
24	1.48/1.19 m		36.39			
25	1.56 m		28.26			
26	0.90 d	6.6	22.46			
27	0.91 d	6.6	22.58			
2,3-acetonide:						
Meβ	1.47 s		28.54	1.47 s		28.54
Meα	1.34 s		26.57	1.34 s		26.57
C	–		108.21	–		108.28
20,22-acetonide:						
Meβ	1.32 s		26.85	–		–
Meα	1.41 s		28.94	–		–
C	–		106.85	–		–

^a^ 800/200 MHz; ^b^ 500/125 MHz; s=singlet; d=doublet; unresolved multiplet.

**Table 2:** ^1^H and ^13^C chemical shifts of the R group in compounds **6** and **7** in CDCl_3_.

Atom no.	7^b^		6^b^	
	H	C	H	C
1′	–	172.83	–	172.82
2′	2.36	34.66	2.33	34.69
3′	1.66	24.64	1.64	24.62
4′	1.34	29.03	1.38-1.29	29.03*
5′	1.36-1.31	29.07*		29.08*
6′		29.08*		29.08*
7′	1.33	29.10		29.10*
8′	1.63	24.96	1.62	24.95
9′	2.30	34.34	2.29	34.33
10′	–	173.92	–	173.91
11′	–	–	–	–
12′	4.04	64.02	4.04	64.01
13′	1.72	26.90	1.73	26.87
14′	2.03	35.79	2.03	35.77
15′	–	133.67	–	133.67
16′	5.13	125.06	5.14	125.03
17′	2.08	26.65	2.07	26.64
18′	1.99	39.66	1.99	39.66
19′	–	135.11	–	135.10
20′	5.15	124.37	5.15	124.35
21′	2.02	28.25*	2.02	28.24*
22′	2.02	28.26*	2.02	28.25*
23′	5.16	124.27	5.15	124.25
24′	–	134.96	–	134.96
25′	1.99	39.74	1.99	39.73
26′	2.08	26.65	2.08	26.64
27′	5.12	124.25	5.12	124.23
28′	–	134.90	–	134.89
29′	1.99	39.72	1.99	39.71
30′	2.07	26.76	2.07	26.74
31′	5.10	124.39	5.10	124.37
32′	–	131.24	–	131.25
33′	1.69	25.68	1.69	25.69
34′	1.60	17.67	1.61	17.66
35′	1.60	15.99	1.61	15.99
36′	1.60	16.04	1.61	16.03
37′	1.60	16.03	1.61	16.02
38′	1.60	15.86	1.61	15.86

^a^800/200 MHz; ^b^500/25 MHz; *tentative assignments.

### Preparation of Self-Assembled Ecdysteroid NPs 6_NP_ and 7_NP_

A 12 mg aliquot of compound **6** or **7** was dissolved in 1.5 ml of acetone (8 mg/ml), and under mild stirring (350 rpm), the prepared solution was added dropwise to a double volume of Milli-Q^®^ (Merck KGaA, Darmstadt, Germany) ultrapure water for 5 min at RT. The spontaneous self-assembly of the bioconjugates occurred immediately, as a consequence of local secondary interactions guided by the hydrophobic squalene chains. Following this, the organic solvent was evaporated at 25°C on a rotary evaporator that afforded the corresponding aq. nanosuspensions **6_NP_** or **7_NP_** in a final concentration of 4 mg/ml.

### Characterization of Nanoparticles

Nano assemblies **6_NP_** and **7_NP_** were initially characterized by transmission electron microscopy (TEM) in order to investigate their morphology. The images were taken by a FEI Tecnai G^2^ 20 X–Twin instrument (FEI Corporate Headquarters, Hillsboro, OR, USA) using 200 kV accelerating voltage. The samples were drop casted on by 200 mesh, copper supported lacey–carbon grids. In order to further investigate the size and colloidal stability of the self–assembled particles in liquid media, dynamic light scattering (DLS) measurements were carried out on a Malvern Zetasizer Nano ZS instrument (Malvern Instruments, Malvern, UK) using disposable folded capillary cells. Particle size distribution (PSD), average hydrodynamic diameter (Z-Average) and polydispersity index (PdI) measurements were performed to assess particle size and dispersity, while zeta potential measurements aimed to determine long-term colloidal stability.

### Cell Lines

Two non-adherent cell lines were used, L5178 mouse T-cell lymphoma (ECACC catalog number 87111908, U.S. FDA, Silver Spring, MD, U.S.), and its multidrug resistant counterpart (L5178_MDR_) that swas established by transfection with pHa MDR1/A retrovirus (Pastan et al., 1988). Cells were cultured in McCoy’s 5A media supplemented with nystatin, L-glutamine, penicillin, streptomycin, and 10% heat-inactivated horse serum, at 37°C and 5% CO_2_. The MDR cell line was selected by culturing the infected cells with 60 ng/L colchicine (Sigma). Media, horse serum, and antibiotics were purchased from Sigma. The human breast cancer cell lines MDA*-*MB-231 and MCF7 were obtained from ECACC (European Collection of Cell Cultures, Salisbury, UK). Cells were grown in Minimum Essential Medium (MEM) supplemented with 10% fetal calf serum, 1% non*-*essential amino acids, and 1% penicillin-streptomycin. All media and supplements for these experiments were obtained from Lonza Group Ltd. (Basel, Switzerland). The cells were maintained at 37*°*C in humidified atmosphere containing 5% CO_2_.

### Evaluation of the ABCB1 Inhibitory Activity of Compounds 3 and 4

ABCB1 inhibitory activity of compounds **3** and **4** was evaluated through the intracellular accumulation of rhodamine 123, an ABCB1 substrate fluorescent dye, by flow cytometry as published before (Martins et al., 2012). Briefly, 2 x 10^6^ cells/ml were treated with 2 or 20 µM of either compound. After 10 min incubation, rhodamine 123 (Sigma-Aldrich) was added to a final concentration of 5.2 μM and the samples were incubated for 20 min at 37°C in water bath. Samples were centrifuged (Heraeus Labofuge 400, Thermo Fisher Scientific, Waltham, MA, USA) (2000 rpm, 2 min) and washed twice with phosphate buffer saline (PBS, Sigma-Aldrich). The final samples were re-suspended in 0.5 ml PBS and its fluorescence measured with a Partec CyFlow flow cytometer (Partec, Münster, Germany). 100 nM of tariquidar, kindly provided by Milica Pešić (Institute for Biological Research Sinisa Stankovic, Belgrade, Serbia), was used as a positive control.

### Evaluation of the Interaction Between Compound 3 or 4 and Doxorubicin or Paclitaxel

The checkerboard microplate method was used to evaluate the combined activity of doxorubicin (Teva, Budapest, Hungary) or paclitaxel (Teva, Budapest, Hungary) and compound **3** and **4** on the cell viability of L5178_MDR_, L5178, MCF-7, and MDA-MB-231 cell lines as described before (Martins et al., 2012).

L5178_MDR_ and L5178cells were incubated at 6,000 cells/well density in presence of doxorubicin and/or compound **3** or **4** in McCoy’s 5A medium (Sigma-Aldrich) for 72 h at 37*°*C, 5% CO_2_. Then, 3-(4,5-dimethylthiazol-2-yl)-2,5-diphenyltetrazolium bromide (MTT, Sigma-Aldrich) was added to each well at a final concentration of 0.5 mg/ml, and after 4 h of incubation, 100 ml of 10% sodium dodecyl sulfate (SDS) (Sigma-Aldrich) in 0.01M HCl was added to each well. The plates were further incubated overnight, and the optical densities were read at 540 and 630 nm using an ELISA reader (Multiskan EX, Thermo Labsystem, Milford, MA, USA). Ecdysteroids and doxorubicin were administered at the 3.125–100 µM, and 67.35 nM–8.62 µM concentration ranges respectively.

Regarding MCF-7 and MDA-MB-231, cells were seeded at 5 x 10^3^ cells/well and incubated overnight for proper cell adhesion. Afterwards, the cells were treated with the tested compounds for 72 h at 37*°*C, and 5% CO_2_. Ecdysteroids were administered at the same concentration range as indicated above, and the concentration ranges for doxorubicin (Dox) and paclitaxel (PCT) were as follows: Dox: 3.9–500 nM (MCF-7), and 7.8 nM–1.0 µM (MDA-MB-231); PCT: 0.54 pM–7.0 nM (MCF-7), and 0.23–30 nM (MDA-MB-231). Following the incubation, 44 µl/well MTT reagent (5 mg/ml in PBS) was added to the samples, and after 4 h of further incubation, the supernatant was replaced with DMSO (100 µl/well) and the plates were shaken for 45 min at 37*°*C to dissolve formazan crystals. The optical density was read at 545 nm wavelength using a microplate reader (Stat Fax-2100, Awareness Technology INC, USA).

In all cases, the interaction was evaluated using the CompuSyn software (CompuSyn Inc., Paramus, NJ, USA) at each constant ratio of compound vs. doxorubicin or compound vs. paclitaxel (M/M), and combination index (CI) values were obtained for 50%, 75%, and 90% of growth inhibition.

### Evaluation of the Interaction Between Nanoassemblies 6_NP_ or 7_NP_ and Paclitaxel on MCF-7 Cells

MCF-7 cells were cultured as described above, and the effect of PCT on cell viability was tested by MTT assay with or without the presence of **6_NP_** or **7_NP_**. Treatment groups were as follows: serial dilutions of PCT (group 1), and serial dilutions of PCT in the presence of a fix concentration of 10 (group 2) or 30 (group 3) µM of **6_NP_** or **7_NP_**. Nanoassemblies were added to the wells directly from their aqueous solution so that they were diluted with medium only, and no DMSO was present in any of the wells. Dose-response curves with respect to PCT were calculated by nonlinear regression using the log inhibitor vs. normalized response model of GraphPad Prism 5 software.

### Evaluation of the Interaction Between Nanoassemblies 6_NP_ or 7_NP_ and Doxorubicin on L5178_MDR_ Cells

Aqueous nanosuspensions (4 mM) of 6_NP_ or 7_NP_ were incubated in 6-times their volume of horse serum (Sigma-Aldrich) for 24 h at 37*°*C, 5% CO_2_, and the resulting mixture was used as the treatment sample. Treatment groups were as follows: serial dilutions of Dox (group 1), and serial dilutions of Dox in the presence of a fix concentration of 37.5 (group 2), 75 (group 3) or 150 (group 4) µM of **6_NP_** or **7_NP_**. Due to the amount of horse serum added to each well in group 3, all wells including cell and solvent controls were substituted with 22.5% of horse serum that did not cause any observable change in the cell growth as compared with the cell control containing 10% of horse serum supplement. In this test, no DMSO was added to any of the wells. Cell viability was evaluated by MTT as described above, and dose-response curves with respect to Dox were calculated by nonlinear regression using the log inhibitor vs. normalized response model of GraphPad Prism 5 software.

## Results and Discussion

### Organic Synthesis

**Scheme 1:**
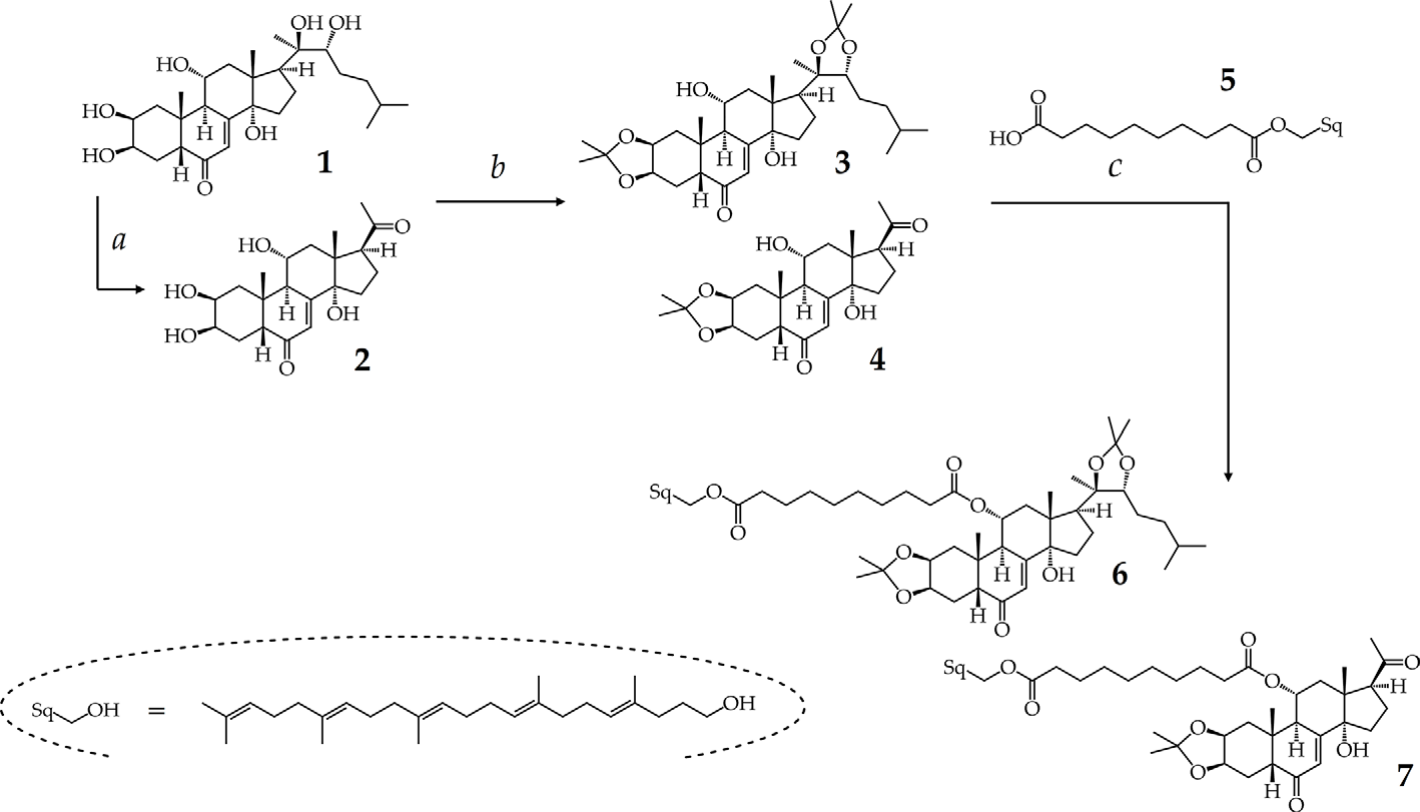
Semi-synthesis of ajugasterone C analogs. Reaction conditions: (a) PIDA (1 equiv.), CH_3_OH, RT, 1 h; (b) PMA hydrate, acetone, RT, 25 min; (c) cpd. 5: sebacic acid (1.25 equiv.), DMAP (2 equiv.), EDC·HCl (3 equiv.), RT, Ar, overnight.

We have previously found that sidechain-cleaved ecdysteroid acetonides may act as similarly potent adjuvant antitumor agents as diacetonides of their parent compounds, but without any direct inhibitory activity on the ABCB1 (also referred to as P-glycoprotein or P-gp) efflux transporter (Hunyadi et al., 2017). To further explore this, our work aiming to prepare 11-squalenoylated ecdysteroids was initiated in two directions: first, we prepared 11α-hydroxypoststerone (**2**) from ajugasterone C (**1**) by oxidative sidechain cleavage using (diacetoxyiodo)benzene (PIDA), a hypervalent iodine(III) reagent that we previously reported to be efficient and selective for this transformation (Issaadi et al., 2019). Compound **2** was obtained in a good yield (74.8%). Both compounds **1** and **2** were then subjected to further transformations as presented in **Scheme 1**.

Previously, we found that dioxolane substitution of ecdysteroids on the vicinal diols, and particularly on the 2,3-diol, is prerequisite of a potent chemo-sensitizing effect (Martins et al., 2013). Therefore, compounds **1** and **2** were converted to their corresponding 2,3;20,22-diacetonide (**3**) or 2,3-acetonide (**4**) analog by phosphomolybdic acid (Martins et al., 2012), which afforded us both derivatives in relatively good yields (**Scheme 1**). These compounds were of particular interest for our study due to their presumed adjuvant antitumor properties, and due to the sterically not hindered 11α-OH group on their structure allowing a convenient coupling of a squalene-derived lipophilic moiety through esterification, and therefore providing a good opportunity for preparing their self-assembling bioconjugate prodrugs.

Since the self-assembly inducer squalene is devoid of a suitable chemical function for the linkage of a drug, its synthetic modification is necessary prior to the coupling (Desmaele et al., 2012). Therefore, we prepared compound **5** following previously published strategies, by first transforming squalene to 1,1′,2-trisnorsqualene alcohol (Maksimenko et al., 2014). Then we conjugated the terminal hydroxyl moiety of this alcohol with sebacic acid, serving as linker, to yield compound **5** whose carboxylic moiety was required for the preparation of esters (Borrelli et al., 2014).

Our preliminary small-scale reactions indicated that the condensation of the inducer (**5**) with ecdysteroid substrates **3** or **4** is chemoselective for the secondary alcohol and allows the synthesis of bioconjugates coupled through the 11α-OH of the ajugasterone C analogs. Following scale-up and chromatographic purification, the conjugates were stored under argon atmosphere at –20°C to ensure their chemical stability until further utilization.

### NMR Investigations

**Figure 1:**
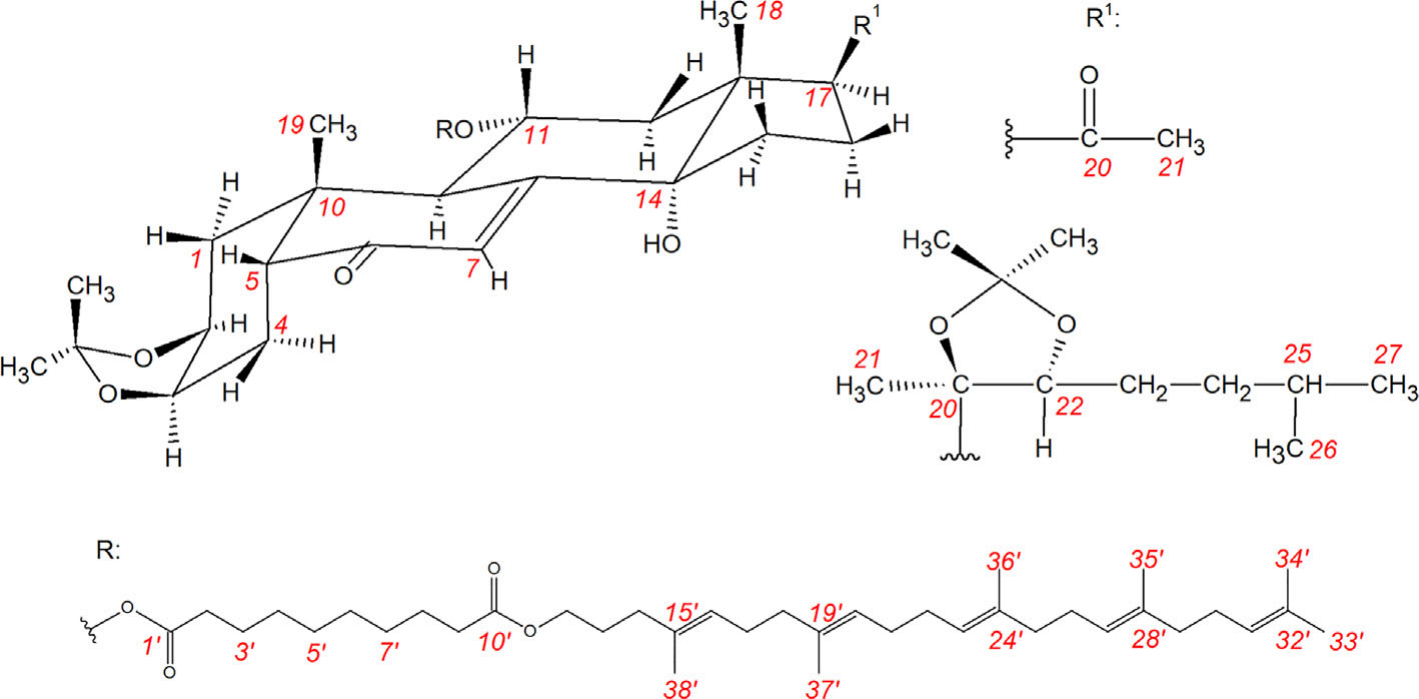
Chemical structures of compounds **6** and **7** and their atomic numbering.

Squalenoylated compounds **6** and **7** were subjected to an in-depth study by high-resolution NMR that allowed us to achieve a complete signal assignment. Chemical structures and ^1^H and ^13^C NMR data for the steroid core of each compound are shown in **Figure 1** and **Tables 1** and **2**, respectively. NMR spectra and the complete signal assignment including that of the ester sidechain coupled at C-11 are presented in detail as **Supplementary Material, Figures S1–S6**.

Squalenoylation is a rapidly emerging strategy to prepare innovative novel nanoconjugates from various bioactive compounds (Arias et al., 2011; Desmaele et al., 2012; Borrelli et al., 2014; Maksimenko et al., 2014; Fumagalli et al., 2017a; Fumagalli et al., 2017b; Rouquette et al., 2019), therefore our NMR results may be applicable and useful to the description and purity evaluation of a wide range of such compounds. For example, in our case, the signal ratio of ca. 5 between the olefin protons of the sidechain (overlapping signals in the range of 5.10–5.16 ppm) and the H-7 (5.89 ppm) was a diagnostic measure of a complete coupling.

### Preparation and Characterization of Self-Assembled NPs

**Figure 2:**
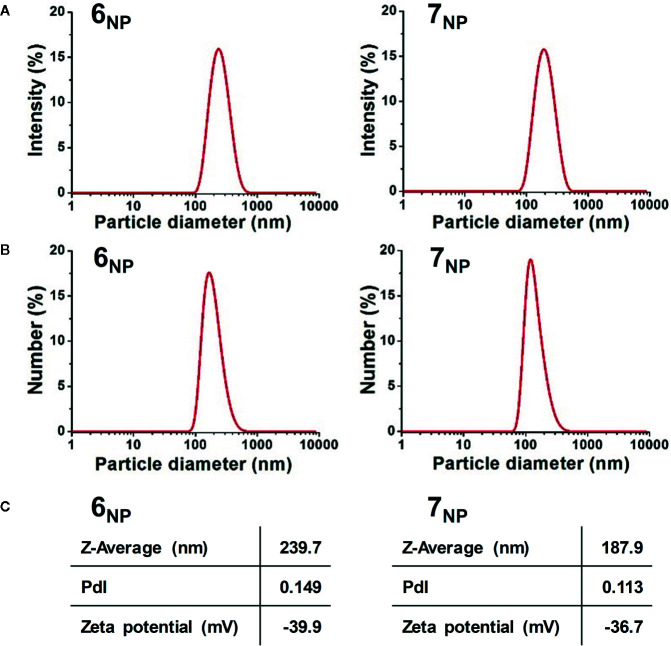
Intensity **(A)** and Number **(B)** particle size distribution graphs, and average hydrodynamic diameter, polydispersity index, and zeta potential values **(C)** of **6_NP_** (left) and **7_NP_** (right).

Following structure elucidation, conjugates **6** and **7** were investigated for their ability to self-assemble to NPs in an aqueous medium. Accordingly, 4 mg/ml stock solutions of **6_NP_** and **7_NP_** were prepared through nanoprecipitation in ultrapure water. Characteristics of the NPs obtained are presented in **Figure 2**.

The room temperature TEM results indicated that neither samples possessed rigid morphology under such experimental conditions. Both **6_NP_** and **7_NP_** had fluid, droplet-like structures (see **Supplementary Material, Figure S7**). The self-assembled droplets could mostly be located at fiber junctions where they could cover the fibers in a wetting manner. The particles assembled from compound **6** appeared similar in size to those of compound **7**, however, as the observed structure of the particles depended heavily on the carbon lace layout, further investigation was necessary.

Unfortunately, our TEM architecture was not suited for cryo imaging, therefore DLS measurements were performed in their place to get a more accurate picture on the size and dispersity, furthermore the colloidal stability of the nanoparticles. The liquid media allowed the observation of these features in a way that is more representative of *in vitro* characteristics. Aliquots of the nanosuspensions were further diluted with water in order to prepare samples for the evaluation of particle size, represented as PSDs by scattered light intensity (**Figure 2A**) and particle number (**Figure 2B**), furthermore for the investigation of the values of Z-Average, PdI, and zeta potential (**Figure 2C**). The Intensity and Number PSD results were in good agreement with one-another for both samples, with slightly shifted mean values that can be explained by the differences between the two representations (Bhattacharjee, 2016). Based on the number distributions, the mean primer particle diameter of **6_NP_** was around 200 nm, while **7_NP_** comprised from particles of around 145 nm. The average hydrodynamic diameters of the samples were 239.7 and 187.9 nm respectively. These results verified the TEM–based observations, as both samples were in comparable size distributions, although **7_NP_** particles were somewhat smaller. The polydispersity index of the samples was quite low (<0.15) indicating that both samples were highly monodispersed. The zeta absolute value of both **6_NP_** and **7_NP_** were well above the theoretical threshold of ±30 mV, therefore their long-term colloidal stability was verified. Additionally, electric repulsion between **6_NP_** particles appeared to be slightly larger than between **7_NP_** particles, although **6_NP_** particles proved larger than the **7_NP_** ones.

The chemical/biological stability of the nanoprecipitated particles in the presence of horse serum was also investigated by DLS. This was to estimate the rate of compounds **6** and **7** getting dissolved into serum lipoproteins when **6_NP_** and **7_NP_** are placed into a lipoprotein-rich medium, so that we can conduct our *in vitro* bioactivity studies on these nanoassemblies in conditions closer to the situation *in vivo* (see section *Interactions of Nanoassemblies **6_NP_** and **7_NP_** With Chemotherapeutics*). To this, a 72-h longitudinal study was performed, throughout which the nanoparticle samples were incubated in horse serum (cell culture supplement in the subsequent *in vitro* studies on L5178_MDR_ cells) with 1:6 volume ratios. At the 0-, 24-, 48-, and 72-h marks, small aliquots were collected from the incubating mixtures and DLS measurements were performed on appropriately diluted (150 ppm for nanoparticles) aqueous dispersions.

**Figure 3:**
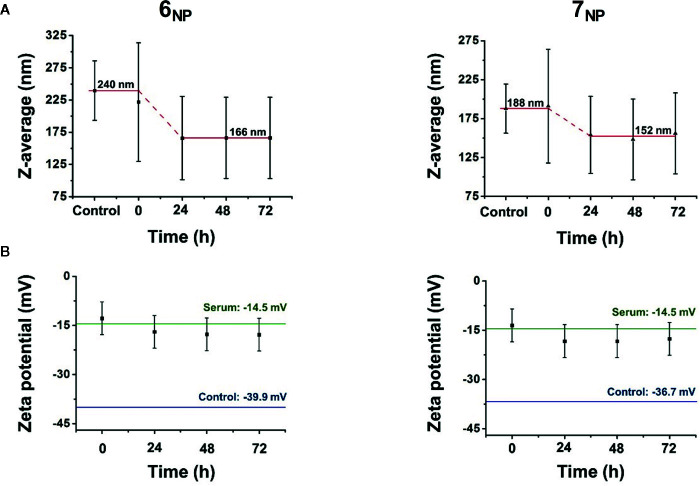
Stability measurements of the self–assembled nanoparticles **6_NP_** and **7_NP_** in horse serum as a function of time according to average hydrodynamic diameter **(A)** and zeta potential **(B)** changes using dynamic light scattering.

Both **6_NP_** and **7_NP_** demonstrated similar behavior according to the results provided by the stability measurements. **Figure 3A** shows the average hydrodynamic diameter of the as–prepared samples, and their size decrease as the function of time due to the presence of horse serum. The Z–average of **6_NP_** particles decreased from 240 nm to 166 nm which is roughly a 31% decrease in radius, while **7_NP_** went down from 188 nm to 152 nm, resulting in a 19% radius decrease. The decomposition of the self–assembled particles happened during the first day of the experiments, after which the particle diameters stabilized, most likely due to the depletion of available macromolecules in the system, which could facilitate particle decomposition. The changes in zeta potential values in **Figure 3B** supported the pervious results. The horizontal lines in the graphs represent the zeta potentials of clean horse medium and the original nanoparticles respectively. While the results did not vary substantially throughout the experiments, a change from 0 to 24 h can be observed in both cases here as well. The initial values corresponding to the 0 h experiments were quite close to the zeta potential of the serum, while the later experiments yielded slightly more negative results, underlining the internal changes within the system.

While hydrodynamic diameter should not be confused with primer article diameter, some assumptions could be made regarding the release of compounds **6** and **7** from their corresponding nanoassemblies. Due to the relatively low standard deviation of the sample sizes, it can be speculated that the decrease in hydrodynamic diameter is proportional to the decrease of primer particle size as well, therefore the radius decreases of 31% and 19% for **6_NP_** and **7_NP_** respectively could be translated into mass, using the expressions of sphere volume (Equation 1) and density (Equation 2). Approximating the self–assembled nanoparticles as ideal spheres with uniform density, and substituting the volume expression into the equation of density (Equation 3), particle radius is directly proportional to the cube root of particle mass (Equation 4):

$$\begin{array}{cc} V_{sphere}=\frac{4}{3}\pi r^{3}\;(1) & \rho =\frac{m}{V}\;(2) \end{array}$$

$$\begin{array}{cc} m=\frac{4}{3}\pi r^{3}\rho \;(3) & m\propto r^{3}\;(4) \end{array}$$

$$6_{\text{NP}}:\;\sqrt[{3}]{0,31}\approx 68\%\;(5)$$

$$7_{\text{NP}}:\;\sqrt[{3}]{0,19}\approx 58\%\;(6)$$

After converting the numbers, **6_NP_** is predicted to release around 68% of its compound **6** contents, while **7_NP_** potentially releases 58% of compound **7** from the self–assembled nanostructures due to the presence of horse serum. Naturally, these numbers are loose estimates at best, however, they could serve as indicators regarding the biological activity of the prepared nanoassemblies.

### Interaction of Compounds 3 and 4 With Chemotherapeutics

Compounds **3** and **4**, parent compounds to **6** and **7**, respectively, were tested on the chemoresistance of five cancer cell lines including a susceptible/resistant cell line pair modeling ABCB1 efflux-mediated multi-drug resistance.

First, efflux pump inhibitory activity of the compounds was assessed. To this, multi-drug resistant L5134_MDR_ mouse lymphoma cells were used, that are transfected to express the human ABCB1 transporter (Pastan et al., 1988). The rhodamine 123 accumulation assay indicated a weak efflux inhibition by compound **3** (inactive at 2 µM, 27.4 ± 1.9% inhibition at 20µM), while compound **4** was inactive (<5% inhibition) at up to 20 µM. This finding was coherent with our previous results on ecdysteroid 2,3;20,22-diacetonides and their sidechain cleaved analogs, namely that removal of the sidechain eliminates efflux pump inhibitory activity (Hunyadi et al., 2017).

Subsequently, the compounds were evaluated on L5134_MDR_ cells for their interaction with doxorubicin, and on MCF-7 and MDA-MB-231 breast cancer cells for their interaction with doxorubicin and paclitaxel. The checkerboard microplate method was used, and the results were evaluated by the Chou-Talalay method (Chou, 2006) as shown in **Table 3**.

**Table 3:** Interaction of compounds **3** and **4** with chemotherapeutics on cancer cell lines at 50%, 75%, and 90% of growth inhibition (ED_50_, ED_75_, and ED_90_, respectively).

Compounds		Drug	CI at						
combined	Cell line	ratio^a^	ED_50_	ED_75_	ED_90_	Dm	m	r	CI_avg_
**3** + Dox	L5178_MDR_	11.6:1	0.09	0.10	0.12	0.71	1.524	0.962	0.11
		23.2:1	0.10	0.13	0.15	1.31	1.470	0.968	0.14
		46.4:1	0.13	0.17	0.23	2.25	1.360	0.979	0.19
**3** + Dox	L5178	75:1	0.40	0.51	0.67	6.97	0.809	0.847	0.57
		150:1	0.88	0.63	0.44	25.27	1.438	0.916	0.58
		300:1	1.22	1.17	1.11	50.82	1.024	0.946	1.15
**3** + Dox	MCF-7	100:1	1.26	0.82	0.60	10.5	2.468	0.942	0.78
		200:1	1.12	1.06	1.16	15.6	1.545	0.995	1.12
		400:1	1.21	1.06	1.07	25.3	2.146	0.998	1.09
**3** + PCT	MCF-7	7,143:1	1.21	1.34	1.59	16.5	1.545	0.967	1.44
		14,285:1	1.27	1.22	1.25	26.5	2.284	0.999	1.24
		28,571:1	1.10	1.00	0.96	31.3	3.206	0.992	1.00
**3** + Dox	MDA-MB-231	100:1	1.43	1.01	1.01	12.8	1.534	0.940	1.08
		200:1	1.09	0.78	0.78	16.9	2.062	0.948	0.83
		400:1	1.10	0.87	0.93	26.5	2.506	0.910	0.94
**3** + PCT	MDA-MB-231	1,667:1	1.71	1.15	1.48	17.4	1.265	0.990	1.41
		3,333:1	1.48	1.40	2.28	24.9	1.180	0.988	1.85
		6,667:1	1.31	1.48	2.48	32.9	1.295	0.987	1.95
**4** + Dox	L5178_MDR_	11.6:1	1.20	0.93	0.72	6.64	1.727	0.991	0.87
		23.2:1	1.09	0.88	0.72	10.9	1.688	0.995	0.83
		46.4:1	1.17	0.93	0.75	19.74	1.822	0.996	0.88
**4** + Dox	L5178	75:1	1.80	1.97	2.17	20.97	2.159	0.964	2.04
		150:1	2.12	2.41	2.80	44.87	1.895	0.977	2.56
		300:1	1.69	2.52	3.84	60.94	1.239	0.923	3.04
**4** + Dox	MCF-7	100:1	1.41	1.45	1.51	9.40	1.426	0.976	1.47
		200:1	1.28	1.07	0.89	16.8	1.958	0.966	1.02
		400:1	1.48	1.67	1.91	37.0	1.246	0.995	1.76
**4** + PCT	MCF-7	7,143:1	2.78	3.08	3.41	21.2	1.608	0.972	3.19
		14,285:1	2.92	3.25	3.61	43.2	1.591	0.954	3.37
		28,571:1	2.38	3.08	3.98	66.9	1.303	0.998	3.41
**4** + Dox	MDA-MB-231	100:1	0.58	0.62	0.78	7.9	1.004	0.996	0.69
		200:1	0.64	0.69	0.92	15.8	1.101	0.992	0.80
		400:1	0.66	0.71	0.94	27.1	1.302	0.976	0.81
**4** + PCT	MDA-MB-231	1,667:1	1.74	1.52	1.33	4.730	1.514	0.997	1.46
		3,333:1	1.58	1.10	0.77	8.536	2.165	1.000	1.02
		6,667:1	1.25	1.29	1.34	13.326	1.214	0.973	1.31

CI, combination index; CI_avg_, weighted average CI value; CI_avg_ = (CI_50_ + 2CI_75_ + 3CI_90_)/6. CI < 1, CI = 1, and CI > 1 represent synergism, additivity, and antagonism, respectively. Dm, m, and r represent antilog of the x-intercept, slope, and linear correlation coefficient of the median-effect plot, respectively. Dox, doxorubicin; PCT, paclitaxel. Strongest interactions observed are highlighted as red (synergism) or blue (antagonism).

a Drug ratios represent molar ratios in each case.

As we expected based on our previous results (Martins et al., 2012), compound **3** exerted strong synergism with doxorubicin on the MDR mouse lymphoma cell line. Somewhat surprisingly, however, compound **3** did not show synergism with doxorubicin on MCF-7 or MDA-MB-231 cells, and it even moderately antagonized the effect of paclitaxel on both breast cancer cell lines. Previously we found that 20-hydroxyecdysone 2,3;20,22-diacetonide (20DA) could decrease cytotoxicity of cisplatin on MCF-7 cells but increased the effect of paclitaxel on the same cell line. This suggests that the additional 11α-OH group and/or the missing 25-OH group (compound **4** vs. 20DA) plays a more important and complex role in the structure-activity relationships than previously thought.

As for compound **4** combined with doxorubicin, no synergism was found on the MDR mouse lymphoma or the MCF-7 cells, but a moderate synergism (CI_avg_ 0.7–0.85) was observed on the triple-negative MDA-MB-231 cell line. Several doxorubicin-containing neoadjuvant chemotherapeutic strategies have shown promise in clinical studies on triple-negative breast cancer (TNBC) (Park et al., 2018; Bergin and Loi, 2019). While the activity of compound **4** is rather weak in this regard, mainly when comparing with that of compound **3** on the MDR lymphoma cell line, it may still be of interest to initiate further studies on ecdysteroid derivatives in combination with doxorubicin on various TNBC models.

It was a most unexpected outcome of our study that compound **4** acted in strong antagonism (CI_avg_>3.3) with paclitaxel, in other words, that it exerted a potent cytoprotective effect against this chemotherapeutic agent on MCF-7 cells. While this makes this compound and, supposedly, its pro-drugs including 7_NP_ completely unsuitable as an adjuvant anticancer agent, it may suggest its possible cytoprotective effect against other stressors.

At this time, it is hard to point out the exact mechanism of action for the above-detailed effects of compounds **3** and **4** on the studied cancer cell lines. The interaction of compound **3** with doxorubicin is clearly connected somehow to the upregulated ABCB1, since it was much weaker on the L5178 cells than on the L5178_MDR_ cells. Further, similarly to our previous results on ecdysteroid 2,3-acetonides, we found that a synergistic interaction does not require that the ecdysteroid acts as a potent efflux inhibitor. It is worth mentioning, however, that the rhodamine123 accumulation is a rapid assay with a 20 min incubation that was designed to assess functional inhibition of the transporter. Therefore, it does not give any information about what happens during the 72 h–long incubation period on the checkerboard plates. To evaluate this, it would be important to conduct further studies on ecdysteroid 2,3-acetonides concerning their potential to interfere with the expression of the pump.

Nevertheless, while the synergistic activity of ecdysteroid acetonides at least with doxorubicin appears to be strongly amplified by upregulated ABCB1, this interaction could also be observed at certain drug-drug ratios on the non-MDR mouse lymphoma, suggesting that ABCB1 overexpression is not a prerequisite for this bioactivity. This fits well with our previous results on other ecdysteroid derivatives that very strongly hypersensitized the non-MDR SH-SY5Y neuroblastoma cell line to vincristine (Müller et al., 2017). As in our previous studies, here we also observed the trend that the strength of interaction depends on the ecdysteroid-chemotherapeutic ratio, and it seems to have a “best ratio” where the interaction is the most significant. It may be of interest in this regard that we recently found 20-hydroxyecdysone (20E; structural isomer of compound **1**, differing in the position of one hydroxyl group) and poststerone (11-deoxy analog of compound **2**) to act on protein kinase B (Akt) with a bell-shaped dose-response curve (Issaadi et al., 2019). Ecdysteroids can modulate Akt phosphorylation in a Ca^2+^-dependent manner (Gorelick-Feldman et al., 2010), and while this was first observed for 20E as an activation, some ecdysteroids may also act as inhibitors (Csábi et al., 2015). Considering the central role of Akt in cell death and survival (Song et al., 2019), one may hypothesize that it should somehow be involved in the chemoresistance altering activity of ecdysteroids. Unfortunately, at this point we have no information about how ecdysteroid acetonides influence this pathway.

### Interactions of Nanoassemblies 6_NP_ and 7_NP_ With Chemotherapeutics

**Figure 4:**
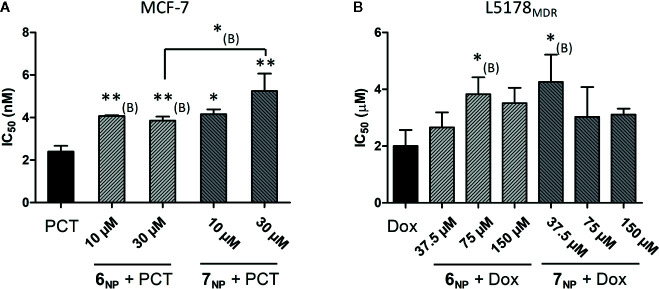
Interaction of **6_NP_** and **7_NP_** with paclitaxel on MCF-7 **(A)** and with doxorubicin on L5178_MDR_ cells **(B)**. The bars show the IC_50_ values of the chemotherapeutic agent with or without the presence of a given fix concentration of **6_NP_** or **7_NP_**, error bars show SEM. For the results shown in **(B)**, the nanosuspensions were preincubated with 6x volume of horse serum for 24 h. * and **: p < 0.05, and p < 0.01, respectively, by one-way ANOVA followed by Dunnett’s post-hoc test as compared with the single treatment; *_(B)_ and **_(B)_: p < 0.05, and p < 0.01, respectively, by one-way ANOVA followed by planned comparisons with the single treatment or with the specified other combination by Bonferroni’s post-hoc test.

To evaluate what relevance the above findings with compounds **3** and **4** may have concerning the bioactivity of their prodrug nanoassemblies (**6_NP_** and **7_NP_**, respectively), we focused subsequent efforts to the two most significant interactions mentioned above. Therefore, we tested **6_NP_** and **7_NP_** in combination with PCT on MCF-7 cells, and in combination with doxorubicin on the L5178_MDR_ cells. First, we tested the chemoresistance modulating activity of **6_NP_** and **7_NP_** by simply adding their aqueous nanosuspensions to the cells. Since neither nanosuspension exerted measurable cytotoxicity on MCF-7 cells, the checkerboard setup could not be used in this case, therefore we selected two fix concentrations (10 and 30 µM) of **6_NP_** and **7_NP_** for testing their effect on the cytotoxicity of paclitaxel; the results are shown in **Figure 4A**. Concerning the MDR mouse lymphoma, cytotoxicity for **6_NP_** was very low but measurable, therefore we first tested it by the checkerboard setup. To our surprise, the interaction with doxorubicin measured this way gave CI_avg_ values in the range of 1.24–1.51 that represents moderate or even stronger antagonism instead of the expected synergism. Considering that this experimental setup might be rather different from the *in vivo* situation where the nanoparticles should get dissolved into serum lipoproteins as individual pro-drug molecules, we then tried to re-evaluate the combination treatment in a possibly more relevant new experiment. To this, horse serum (that is the supplement used for the mouse lymphoma cells) was first added to the nanoparticles in a 6:1 ratio, the mixture was incubated for 24 h (that was the plateau of the decrease in NP size, see section *Preparation and Characterization of Self-Assembled NPs*), and this was then further tested on the doxorubicin resistance of L5178_MDR_ cells. Again, the checkerboard setup was ruled out due to the non-measurable cytotoxicity of the horse serum-pretreated **6_NP_** and **7_NP_**, therefore fix concentrations of these were tested; results are shown in **Figure 4B**.

It is clear from our results that both **6_NP_** and **7_NP_**, similarly to their corresponding parent compounds **3** and **4**, significantly protected the MCF-7 cells from the cytotoxic effect of paclitaxel. Further, the relative potency of the two nanoassemblies seems to correlate with that of compounds **3** and **4** (i.e. 4 > 3 and **7_NP_** > **6_NP_**), which would support the assumption that the nanoparticles indeed worked as prodrugs of the 11α-hydroxylated parent ecdysteroids 3 and 4. On the MDR mouse lymphoma cell line, however, a surprising opposite effect was observed for 3 and **6_NP_**: in contrast with the strong synergism between compound **3** and doxorubicin, **6_NP_** significantly decreased the efficacy of the Dox, regardless of the fact that a large fraction of **6_NP_** were pre-dissolved in horse serum to liberate compound **6** in its free form (see **Figure 3** and eq. 5 and 6). Therefore, this nanoassembly cannot deliver compound **3** into L5178_MDR_ cells as a prodrug. A very likely explanation to this is connected to the endocytosis of lipoproteins (Zanoni et al., 2018) in which the squalenoylated prodrug is dissolved. Because of this, the prodrug ends up in the acidic (pH ca. 4.5) environment of lysosomes (Hu et al., 2015). This may already be enough to cleave the acid sensitive 2,3-acetonide group (Martins et al., 2012) that is of major importance to a strong chemosensitizing effect of ecdysteroids (Martins et al., 2013). Therefore, it appears that instead of the adjuvant antitumor ecdysteroid diacetonide **3**, the 20,22-monoacetonide, or, to some extent, even the parent compound ajugasterone C (**1**) gets eventually released from compound **6** after treatment by 6_NP_. Similarly, this should also be happening with **7_NP_** that, after internalization, likely releases compound **2** instead of 4. It is not without precedence that an ecdysteroid and its diacetonide derivative act in an opposite way on the chemoresistance of tumor cells: we previously found 20-hydroxyecdysone (20E) to act in antagonism with doxorubicin on L5178_MDR_ cells, in contrast with the strong synergism observed for 20E 2,3;20,22-diacetonide (Martins et al., 2012).

The above findings have two major implications. First, the strategy of using squalenoylated ecdysteroid nanoparticles as adjuvant antitumor agents appears to make it a must to replace the 2,3-acetonide moiety with an acid-resistant alternative. Our current results also suggest that our previous success in using this strategy (Fumagalli et al., 2018) may have been connected to the release of 20E 20,22-monoacetonide that is less potent than the diacetonide but still active in decreasing tumor resistance (Martins et al., 2012). If it is indeed the case, that would mean that with an appropriate acid-resistant 2,3-substituent the previously observed antitumor action could be further improved.

Second, while the cytoprotective nature of the herein reported squalenoylated ecdysteroid acetonide nanoparticles makes them certainly not useful against cancer, it points towards a possible new strategy to deliver adaptogen ecdysteroids to target tissues with an aim to increase stress resistance. Above cancer, LDL-R is upregulated in some healthy tissues and organs; a most obvious example for this is the liver (van de Sluis et al., 2017) but LDL-R also plays an important role in the blood-brain barrier (BBB) (Dehouck et al., 1994) and can be exploited for facilitating transport across the BBB (Molino et al., 2017). As such, squalene conjugation offers a means of targeting in many problematic pathologies other than cancer. Therefore, the observed activity of compounds **4**, **6_NP,_** and **7_NP_** suggests further studies on relevant, preferably *in vivo* pharmacological models to critically evaluate any possible therapeutic potential of the current findings.

## Conclusions

In this work, two new squalenoylated ecdysteroid pro-drugs were prepared, fully characterized, and formulated to self-assembling nanoparticles. In both cases, the successful submicron assembly of compounds **6** (derived from compound **3**) and **7** (from compound **4**) was achieved into **6_NP_** and **7_NP_** respectively. Both **6_NP_** and **7_NP_** formed monodispersed and stable suspensions consisting of fluid, droplet–like particles in a similar size range. Based on DLS results, **6_NP_** is expected to possess slightly greater colloidal stability, although the *in vivo* behavior of the compounds might vary substantially and requires further investigation. The employed functionalization of the ecdysteroids **3** and **4** allows their targeting to tissues containing high levels of LDL-receptor. Compound **3** strongly potentiated the antitumor action of doxorubicin on an MDR lymphoma cell line, while compound **4** was a potent cytoprotective agent strongly antagonizing the effect of paclitaxel on MCF–7 cells. Surprisingly, however, both nanoassemblies **6_NP_** and **7_NP_** antagonized the chemotherapeutic agents’ effect on the studied cancer cell lines. This happens most likely due to the effect of lysosomes resulting in the acid-catalyzed release of the 20,22-monoacetonide and/or the non-substituted form of compound **1** and compound **2** from **6_NP_** and **7_NP_**, respectively. We may conclude that i) exploiting the adjuvant antitumor action of squalenoylated ecdysteroid nanoassemblies requires the design and synthesis of appropriate acid-resistant 2,3-substituents instead of the acetonide group, and ii) the herein reported nanoparticles have no antitumor potential but they might be promising cytoprotective agents targeting LDL-R upregulated tissues such as for example the liver or the blood-brain barrier.

## Data Availability Statement

The raw data supporting the conclusions of this article will be made available by the authors, without undue reservation, to any qualified researcher.

## Author Contributions

MV performed chemical synthesis and purification, prepared nano-assemblies, and wrote first draft of the manuscript. MV and PB characterized nanoparticles. DB, TG, and GT performed NMR spectroscopic studies, and analyzed and interpreted all related data. MN, GS, and AL performed studies on the mouse lymphoma cell lines and analyzed related data. AL performed studies on human gynecological cell lines and analyzed related data. IZ supervised studies on human gynecological cell lines. ZK supervised characterization of nanoparticles. AH conceptualized the study, supervised chemical part of the work, calculated combination indices, designed combination studies on the nanoparticles, and finalized the manuscript. All authors contributed to the article and approved the submitted version.

## Funding

This work was funded by the National Research, Development and Innovation Office, Hungary (NKFIH; K119770), the Ministry of Human Capacities, Hungary grant 20391-3/2018/FEKUSTRAT, and the EU-funded Hungarian grant EFOP-3.6.1-16-2016-00008. MV was supported by the National Talent Program of the Ministry of Human Capacities, Hungary. Publication of the manuscript was supported by the University of Szeged Open Access Fund. The funders had no role in the design of the study; in the collection, analyses, or interpretation of data; in the writing of the manuscript, or in the decision to publish the results.

## Conflict of Interest

The authors declare that the research was conducted in the absence of any commercial or financial relationships that could be construed as a potential conflict of interest.

## References

[B1] Arias J. L. Reddy L. H. Othman M. Gillet B. Desmaële D. Zouhiri F. (2011). Squalene Based Nanocomposites: A New Platform for the Design of Multifunctional Pharmaceutical Theragnostics. ACS Nano 5, 1513–1521. 10.1021/nn1034197 21275408

[B2] Bergin A. R. T. Loi S. (2019). Triple-negative breast cancer: recent treatment advances. F1000Res 8. F1000 Faculty Rev-1342. 10.12688/f1000research.18888.1 PMC6681627 31448088

[B3] Bhattacharjee S. (2016). DLS and zeta potential – What they are and what they are not? J. Controlled Release 235, 337–351. 10.1016/j.jconrel.2016.06.017 27297779

[B4] Bildstein L. Dubernet C. Couvreur P. (2011). Prodrug-based intracellular delivery of anticancer agents. Adv. Drug Delivery Rev. 63, 3–23. 10.1016/j.addr.2010.12.005 21237228

[B5] Bogdan D. Haessner R. Vagvolgyi M. Passarella D. Hunyadi A. Gati T. (2018). Stereochemistry and complete (1) H and (13) C NMR signal assignment of C-20-oxime derivatives of posterone 2,3-acetonide in solution state. Magn. Reson. Chem. 56, 859–866. 10.1002/mrc.4750 29775488

[B6] Borrelli S. Christodoulou M. S. Ficarra I. Silvani A. Cappelletti G. Cartelli D. (2014). New class of squalene-based releasable nanoassemblies of paclitaxel, podophyllotoxin, camptothecin and epothilone A. Eur. J. Med. Chem. 85, 179–190. 10.1016/j.ejmech.2014.07.035 25084144

[B7] Cheetham A. G. Chakroun R. W. Ma W. Cui H. (2017). Self-assembling prodrugs. Chem. Soc. Rev. 46, 6638–6663. 10.1039/C7CS00521K 29019492 PMC5844511

[B8] Chou T. C. (2006). Theoretical basis, experimental design, and computerized simulation of synergism and antagonism in drug combination studies. Pharmacol. Rev. 58, 621–681. 10.1124/pr.58.3.10 16968952

[B9] Csábi J. Hsieh T.-J. Hasanpour F. Martins A. Kele Z. Gáti T. (2015). Oxidized Metabolites of 20-Hydroxyecdysone and Their Activity on Skeletal Muscle Cells: Preparation of a Pair of Desmotropes with Opposite Bioactivities. J. Natural Prod. 78, 2339–2345. 10.1021/acs.jnatprod.5b00249 26465254

[B10] Dehouck B. Dehouck M. P. Fruchart J. C. Cecchelli R. (1994). Upregulation of the low density lipoprotein receptor at the blood-brain barrier: intercommunications between brain capillary endothelial cells and astrocytes. J. Cell Biol. 126, 465–473. 10.1083/jcb.126.2.465 8034745 PMC2200038

[B11] Delplace V. Couvreur P. Nicolas J. (2014). Recent trends in the design of anticancer polymer prodrug nanocarriers. Polym. Chem. 5, 1529–1544. 10.1039/C3PY01384G

[B12] Desmaele D. Gref R. Couvreur P. (2012). Squalenoylation: a generic platform for nanoparticular drug delivery. J. Control Release 161, 609–618. 10.1016/j.jconrel.2011.07.038 21840355

[B13] Dinan L. Mamadalieva N. Z. Lafont R. (2020). “Dietary Phytoecdysteroids”, in Handbook of Dietary Phytochemicals. Eds. Xiao J. Sarker S. D. Asakawa Y. (Singapore: Springer), 1–54. 10.1007/978-981-13-1745-3_35-1

[B14] Duddeck H. Dietrich W. Tóth G. (1998). Structure Elucidation by Modern NMR. (Steinkopff, Heidelberg). 10.1007/978-3-642-88310-1

[B15] Fumagalli G. Marucci C. Christodoulou M. S. Stella B. Dosio F. Passarella D. (2016). Self-assembly drug conjugates for anticancer treatment. Drug Discov. Today 21, 1321–1329. 10.1016/j.drudis.2016.06.018 27329268

[B16] Fumagalli G. Stella B. Pastushenko I. Ricci F. Christodoulou M. S. Damia G. (2017a). Heteronanoparticles by self-Assembly of Doxorubicin and Cyclopamine Conjugates. ACS Med. Chem. Lett. 8, 953–957. 10.1021/acsmedchemlett.7b00262 28947943 PMC5601370

[B17] Fumagalli G. Christodoulou M. S. Riva B. Revuelta I. Marucci C. Collico V. (2017b). Self-assembled 4-(1,2-diphenylbut-1-en-1-yl)aniline based nanoparticles: podophyllotoxin and aloin as building blocks. Org. Biomol. Chem. 15, 1106–1109. 10.1039/C6OB02591A 28093593

[B18] Fumagalli G. Giorgi G. Vagvolgyi M. Colombo E. Christodoulou M. S. Collico V. (2018). Heteronanoparticles by Self-Assembly of Ecdysteroid and Doxorubicin Conjugates to Overcome Cancer Resistance. ACS Med. Chem. Lett. 9, 468–471. 10.1021/acsmedchemlett.8b00078 29795761 PMC5949839

[B19] Gorelick-Feldman J. Cohick W. Raskin I. (2010). Ecdysteroids elicit a rapid Ca2+ flux leading to Akt activation and increased protein synthesis in skeletal muscle cells. Steroids 75, 632–637. 10.1016/j.steroids.2010.03.008 20363237 PMC3815456

[B20] Hu Y.-B. Dammer E. B. Ren R.-J. Wang G. (2015). The endosomal-lysosomal system: from acidification and cargo sorting to neurodegeneration. Transl. Neurodegener. 4, 18–18. 10.1186/s40035-015-0041-1 26448863 PMC4596472

[B21] Hunyadi A. Gergely A. Simon A. Toth G. Veress G. Bathori M. (2007). Preparative-scale chromatography of ecdysteroids of Serratula wolffli andrae. J. Chromatogr. Sci. 45, 76–86. 10.1093/chromsci/45.2.76 17425136

[B22] Hunyadi A. Csabi J. Martins A. Molnar J. Balazs A. Toth G. (2017). Backstabbing P-gp: Side-Chain Cleaved Ecdysteroid 2,3-Dioxolanes Hyper-Sensitize MDR Cancer Cells to Doxorubicin without Efflux Inhibition. Molecules 22, 199. 10.3390/molecules22020199 PMC6155823 28125071

[B23] Issaadi H. M. Csábi J. Hsieh T.-J. Gáti T. Tóth G. Hunyadi A. (2019). Side-chain cleaved phytoecdysteroid metabolites as activators of protein kinase B. Bioorg. Chem. 82, 405–413. 10.1016/j.bioorg.2018.10.049 30428419

[B24] Lafont R. Dinan L. (2003). Practical uses for ecdysteroids in mammals including humans: an update. J. Insect Sci. 3, 7. 10.1673/031.003.0701 15844229 PMC524647

[B25] Li Z. Tan S. Li S. Shen Q. Wang K. (2017). Cancer drug delivery in the nano era: An overview and perspectives (Review). Oncol. Rep. 38, 611–624. 10.3892/or.2017.5718 28627697 PMC5562049

[B26] Maksimenko A. Dosio F. Mougin J. Ferrero A. Wack S. Reddy L. H. (2014). A unique squalenoylated and nonpegylated doxorubicin nanomedicine with systemic long-circulating properties and anticancer activity. Proc. Natl. Acad. Sci. U. S. A. 111, E217–E226. 10.1073/pnas.1313459110 24385587 PMC3896189

[B27] Martins A. Toth N. Vanyolos A. Beni Z. Zupko I. Molnar J. (2012). Significant activity of ecdysteroids on the resistance to doxorubicin in mammalian cancer cells expressing the human ABCB1 transporter. J. Med. Chem. 55, 5034–5043. 10.1021/jm300424n 22578055

[B28] Martins A. Csabi J. Balazs A. Kitka D. Amaral L. Molnar J. (2013). Synthesis and structure-activity relationships of novel ecdysteroid dioxolanes as MDR modulators in cancer. Molecules 18, 15255–15275. 10.3390/molecules181215255 24335576 PMC6269874

[B29] Martins A. Sipos P. Der K. Csabi J. Miklos W. Berger W. (2015). Ecdysteroids sensitize MDR and non-MDR cancer cell lines to doxorubicin, paclitaxel, and vincristine but tend to protect them from cisplatin. BioMed. Res. Int. 2015, 895360. 10.1155/2015/895360 26075272 PMC4449901

[B30] Molino Y. David M. Varini K. Jabes F. Gaudin N. Fortoul A. (2017). Use of LDL receptor-targeting peptide vectors for in vitro and in vivo cargo transport across the blood-brain barrier. FASEB J. 31, 1807–1827. 10.1096/fj.201600827R 28108572

[B31] Müller J. Martins A. Csábi J. Fenyvesi F. Könczöl Á. Hunyadi A. (2017). BBB penetration-targeting physicochemical lead selection: Ecdysteroids as chemo-sensitizers against CNS tumors. Eur. J. Pharm. Sci. 96, 571–577. 10.1016/j.ejps.2016.10.034 27810561

[B32] Park J. H. Ahn J.-H. Kim S.-B. (2018). How shall we treat early triple-negative breast cancer (TNBC): from the current standard to upcoming immuno-molecular strategies. ESMO Open 3, e000357. 10.1136/esmoopen-2018-000357 29765774 PMC5950702

[B33] Pastan I. Gottesman M. M. Ueda K. Lovelace E. Rutherford A. V. Willingham M. C. (1988). A retrovirus carrying an MDR1 cDNA confers multidrug resistance and polarized expression of P-glycoprotein in MDCK cells. Proc. Natl. Acad. Sci. 85, 4486. 10.1073/pnas.85.12.4486 2898143 PMC280455

[B34] Pretsch E. Tóth G. Munk E. M. Badertscher M. (2002). Computer-Aided Structure Elucidation (Weinheim, Germany: Wiley-VCH).

[B35] Rouquette M. Lepetre-Mouelhi S. Dufrancais O. Yang X. Mougin J. Pieters G. (2019). Squalene-Adenosine Nanoparticles: Ligands of Adenosine Receptors or Adenosine Prodrug? J. Pharmacol. Exp. Ther. 369, 144–151. 10.1124/jpet.118.254961 30670479

[B36] Shi J. Kantoff P. W. Wooster R. Farokhzad O. C. (2017). Cancer nanomedicine: progress, challenges and opportunities. Nat. Rev. Cancer 17, 20–37. 10.1038/nrc.2016.108 27834398 PMC5575742

[B37] Sobot D. Mura S. Yesylevskyy S. O. Dalbin L. Cayre F. Bort G. (2017). Conjugation of squalene to gemcitabine as unique approach exploiting endogenous lipoproteins for drug delivery. Nat. Commun. 8, 15678. 10.1038/ncomms15678 28555624 PMC5459998

[B38] Song M. Bode A. M. Dong Z. Lee M.-H. (2019). AKT as a Therapeutic Target for Cancer. Cancer Res. 79 (6), 1019–1031. 10.1158/0008-5472.CAN-18-2738 30808672

[B39] van de Sluis B. Wijers M. Herz J. (2017). News on the molecular regulation and function of hepatic low-density lipoprotein receptor and LDLR-related protein 1. Curr. Opin. Lipidol. 28, 241–247. 10.1097/MOL.0000000000000411 28301372 PMC5482905

[B40] Xu X. Ho W. Zhang X. Bertrand N. Farokhzad O. (2015). Cancer nanomedicine: from targeted delivery to combination therapy. Trends Mol. Med. 21, 223–232. 10.1016/j.molmed.2015.01.001 25656384 PMC4385479

[B41] Zanoni P. Velagapudi S. Yalcinkaya M. Rohrer L. von Eckardstein A. (2018). Endocytosis of lipoproteins. Atherosclerosis 275, 273–295. 10.1016/j.atherosclerosis.2018.06.881 29980055

