# Supplementary material for: Squalenoylated Nanoparticle Pro-Drugs of Adjuvant Antitumor 11α-Hydroxyecdysteroid 2,3-Acetonides Act as Cytoprotective Agents Against Doxorubicin and Paclitaxel
Source: Front Pharmacol. 2020 Sep 11;11:552088. doi: 10.3389/fphar.2020.552088 (PMC7516204; doi:10.3389/fphar.2020.552088)
Supplement: Supplementary file 1 [file DataSheet_1.docx]

Supplementary Material

Squalenoylated nanoparticle pro-drugs of adjuvant antitumor 11α-hydroxyecdysteroid 2,3-acetonides act as cytoprotective agents against doxorubicin and paclitaxel

Máté Vágvölgyi^1^, Péter Bélteky^2^, Dóra Bogdán^3,4^, Márta Nové^5^, Gabriella Spengler^5^, Ahmed D. Latif^6,a^, István Zupkó^6,7^, Tamás Gáti^8^, Gábor Tóth^9^, Zoltán Kónya^2,10^, and Attila Hunyadi^1,7,*^

^1^Institute of Pharmacognosy, Interdisciplinary Excellence Centre, University of Szeged, Szeged, Hungary

^2^Department of Applied and Environmental Chemistry, Interdisciplinary Excellence Centre, University of Szeged, Szeged, Hungary

^3^Department of Organic Chemistry, Semmelweis University, Budapest, Hungary

^4^Institute of Materials and Environmental Chemistry, Research Centre for Natural Sciences, Budapest, Hungary

^5^Department of Medical Microbiology and Immunobiology, University of Szeged, Szeged, Hungary

^6^Department of Pharmacodynamics and Biopharmacy, Faculty of Pharmacy, University of Szeged, Szeged, Hungary

^7^Interdisciplinary Centre of Natural Products, University of Szeged, Szeged, Hungary

^8^Servier Research Institute of Medicinal Chemistry (SRIMC), Budapest, Hungary

^9^NMR Group, Department of Inorganic and Analytical Chemistry, Budapest University of Technology and Economics, Budapest, Hungary

^10^MTA-SZTE Reaction Kinetics and Surface Chemistry Research Group, University of Szeged, Szeged, Hungary

^a^on leave from Department of Pharmacology and Toxicology, Faculty of Medicine, Wasit University, Wasit, Iraq.

*** Correspondence:**Attila Hunyadi; [hunyadi.a@pharm.u-szeged.hu](mailto:hunyadi.a@pharm.u-szeged.hu); Tel.: +36-62-546-456

**Table of contents**

[**Figure S1.** ^1^H NMR [800 MHz, CDCl_3_] of compound **7** 2](#_Toc46224307)

[**Figure S2.** ^13^C NMR [200 MHz, CDCl_3_] of compound **7** 3](#_Toc46224308)

[**Figure S3.** edHSQC sections [800/200 MHz, CDCl_3_] of compound **7** 4](#_Toc46224309)

[**Figure S4.** Band-selective HSQC (section: =CH area) of compound **7** 5](#_Toc46224310)

[**Figure S5.** ^1^H NMR [500 MHz] of compound **6** 6](#_Toc46224311)

[**Figure S6.** DEPTQ NMR [500 MHz] of compound **6** 7](#_Toc46224312)

[**Figure S7.** Transmission electron microscope images of **6_NP_** and **7_NP_**. 8](#_Toc46224313)


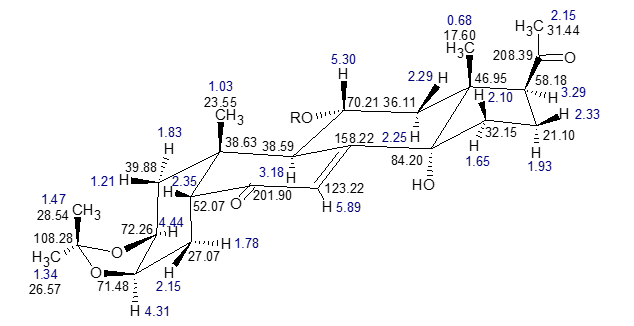


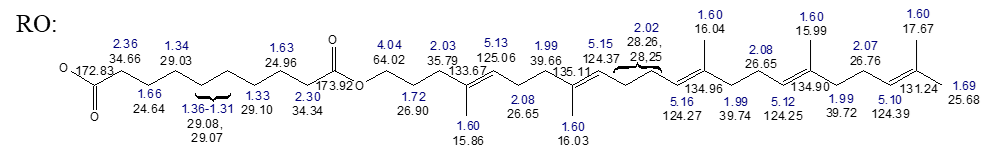


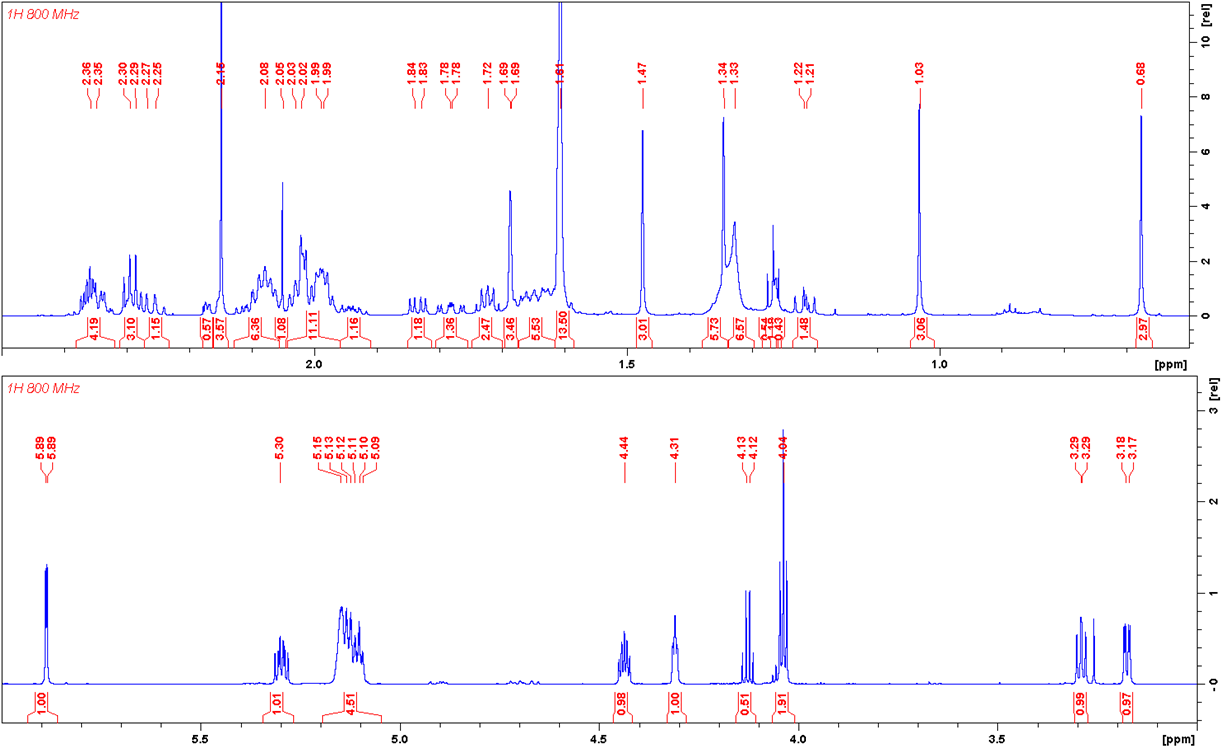


# **Figure S1.** ^1^H NMR [800 MHz, CDCl_3_] of compound **7**


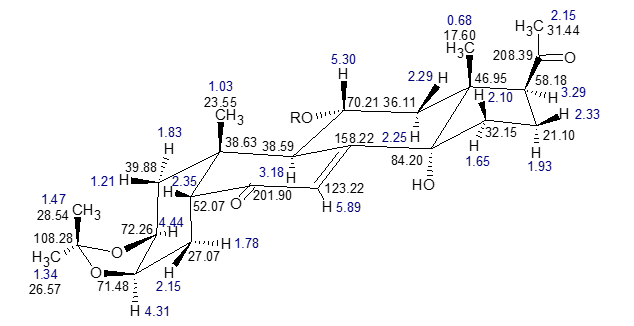


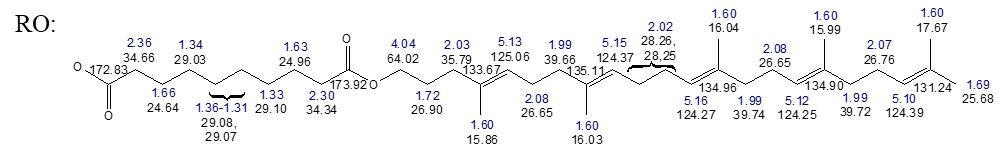


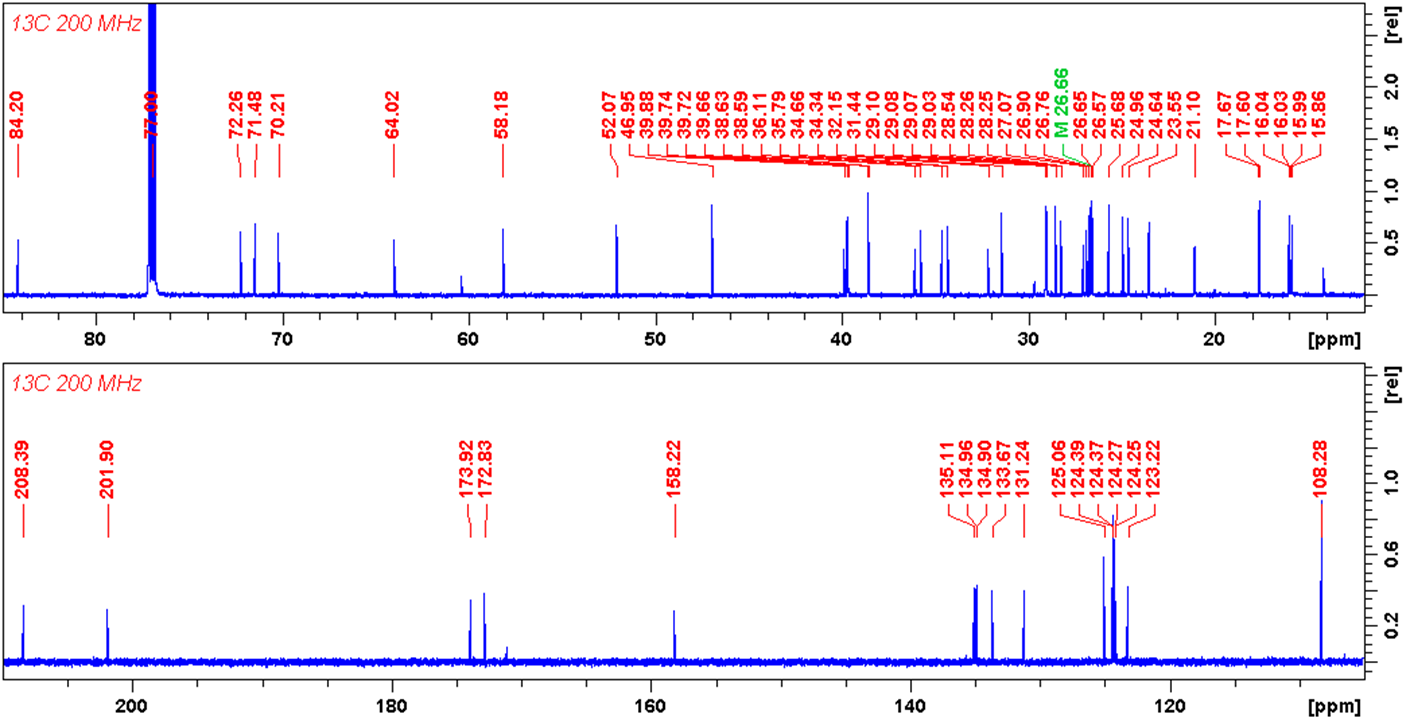


# **Figure S2.** ^13^C NMR [200 MHz, CDCl_3_] of compound **7**


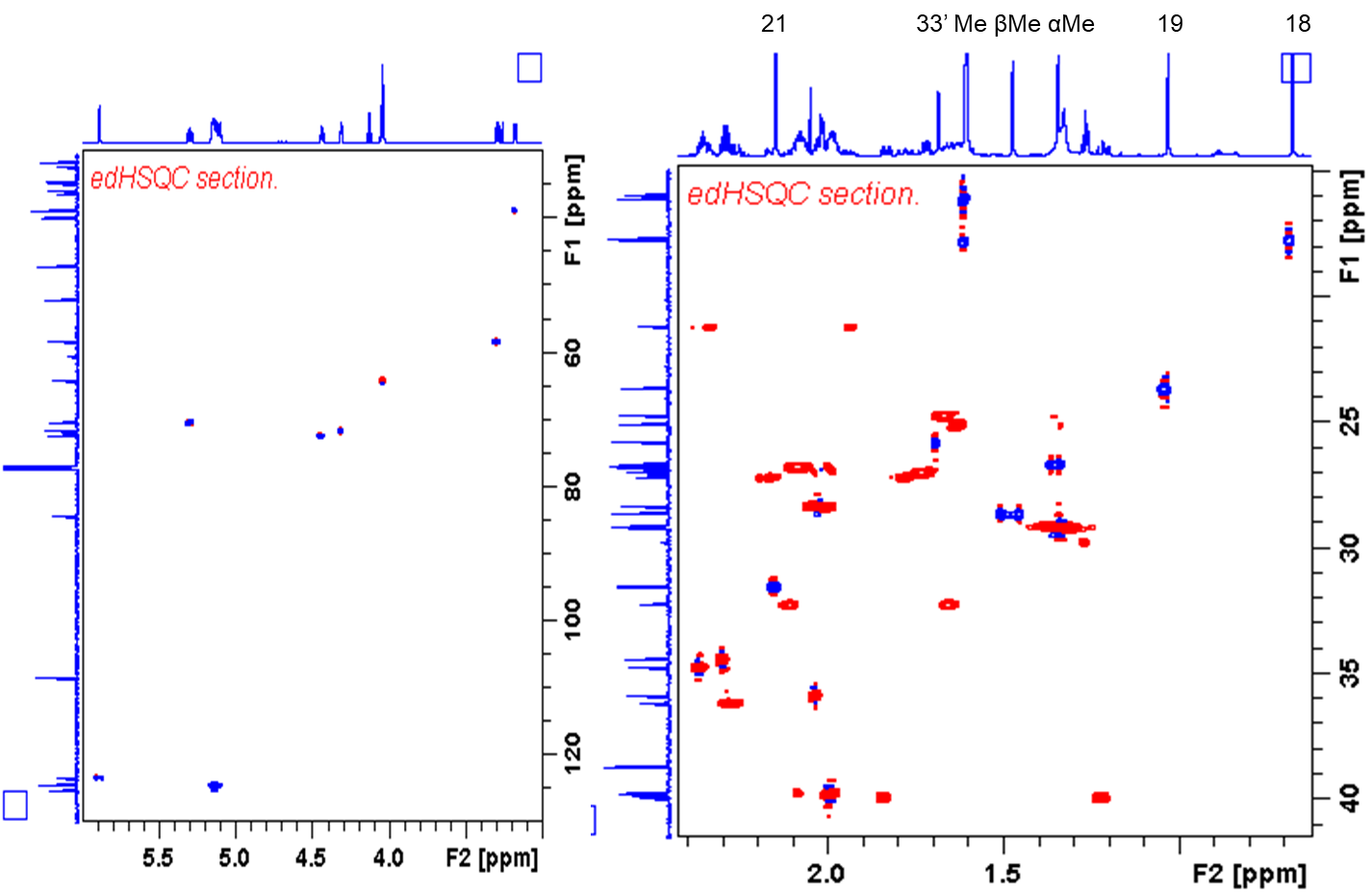


# **Figure S3.** edHSQC sections [800/200 MHz, CDCl_3_] of compound **7**


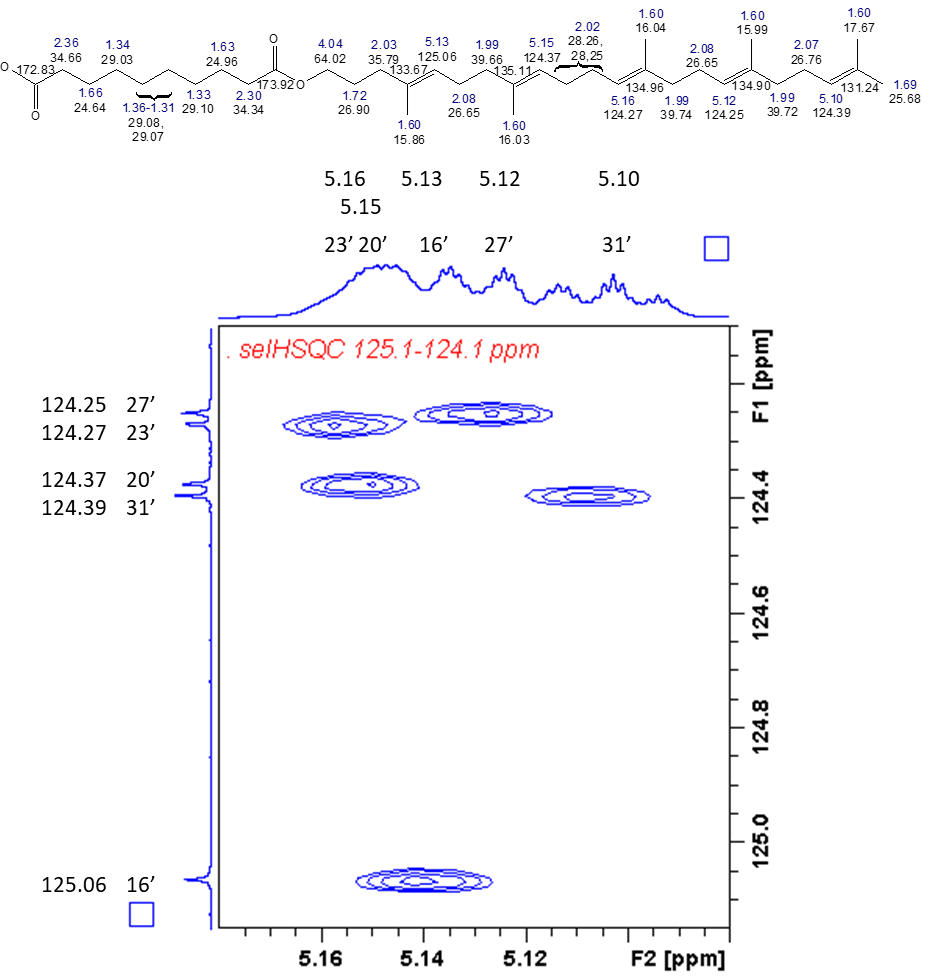


# **Figure S4.** Band-selective HSQC (section: =CH area) of compound **7**


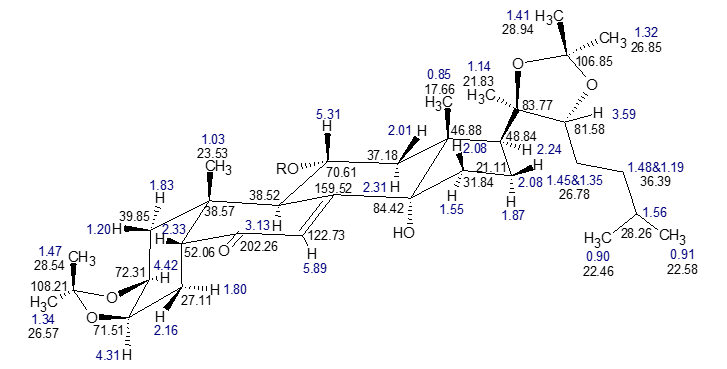


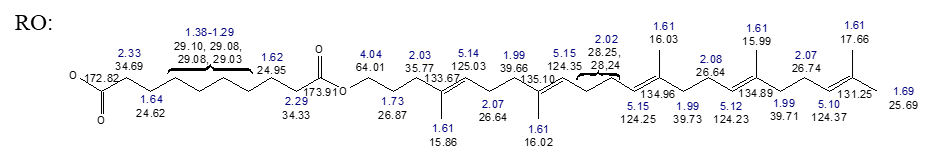


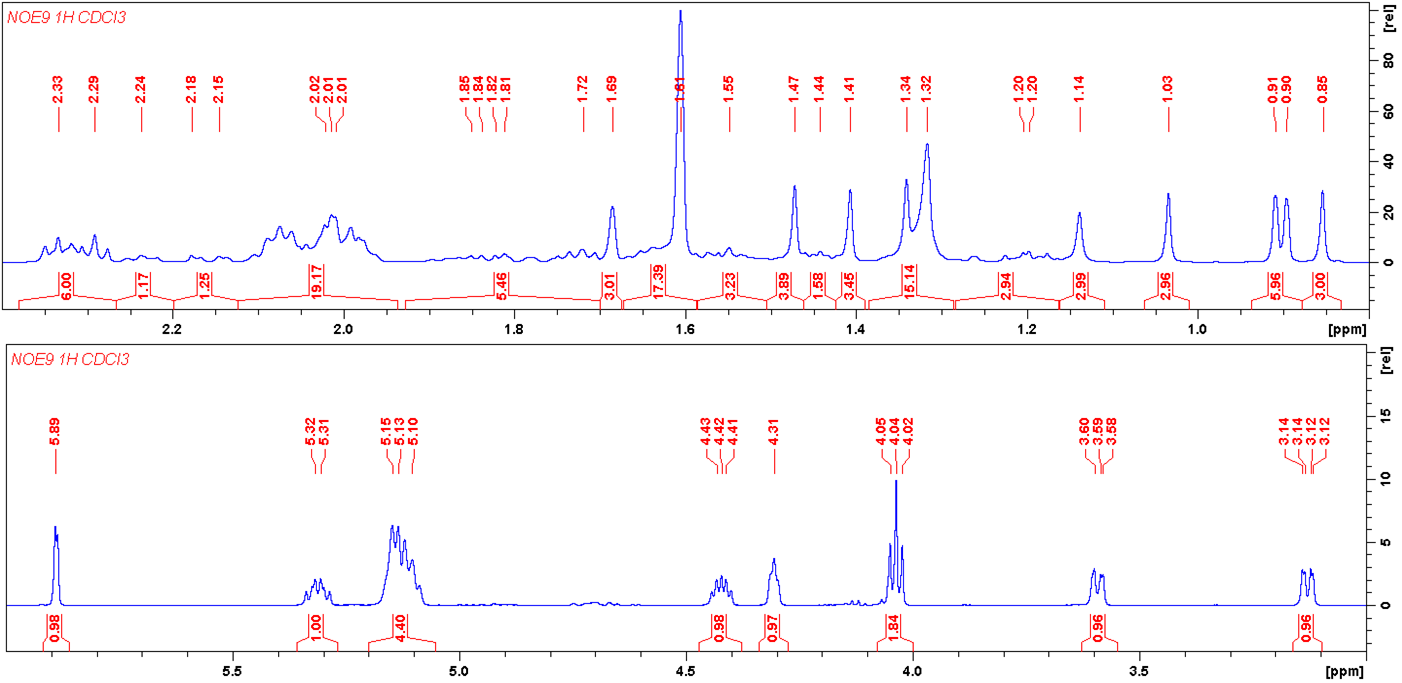


# **Figure S5.** ^1^H NMR [500 MHz] of compound **6**

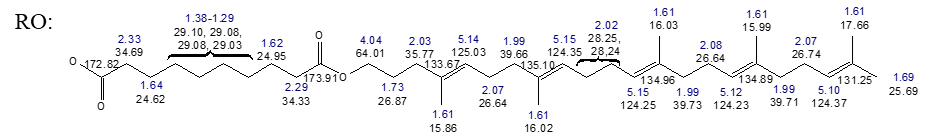


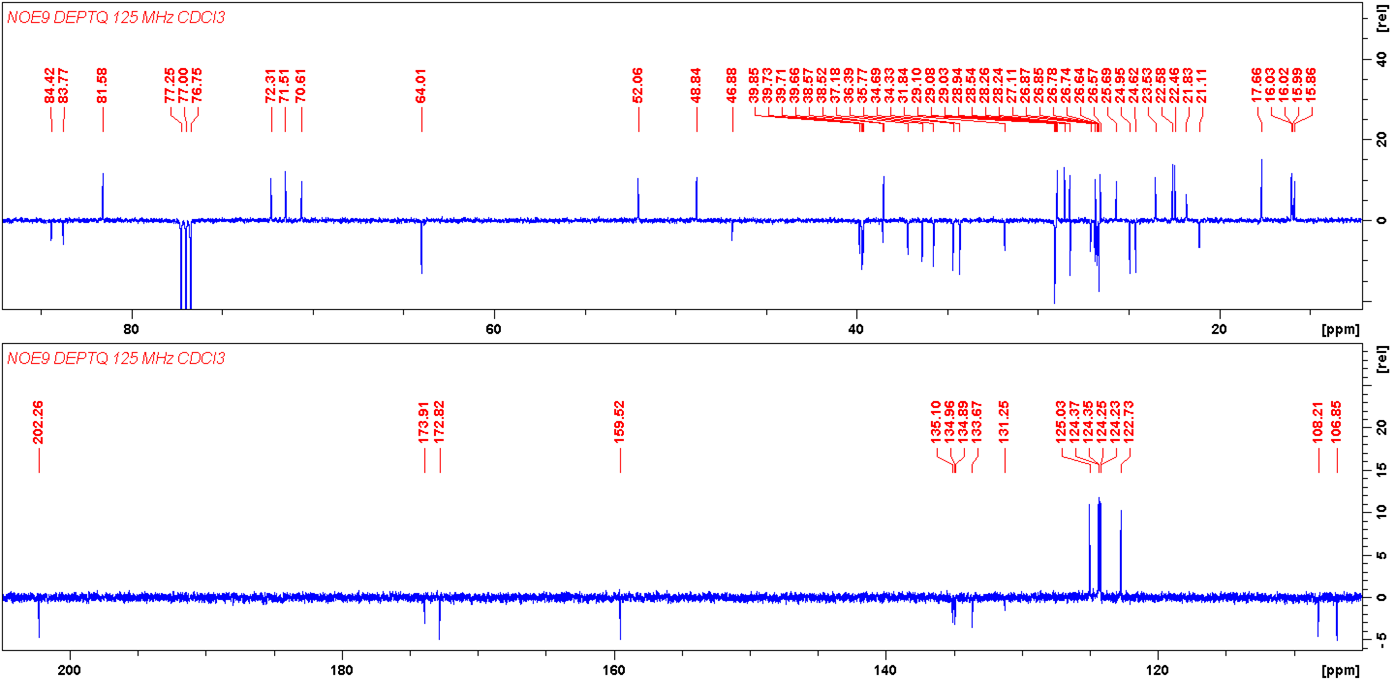


# **Figure S6.** DEPTQ NMR [500 MHz] of compound **6**

# **Figure S7.** Transmission electron microscope images of **6_NP_** and **7_NP_**.
